# Engineering cyanobacteria as a new platform for producing taxol precursors directly from carbon dioxide

**DOI:** 10.1186/s13068-024-02555-9

**Published:** 2024-07-16

**Authors:** Jialing Zhong, Yushu Wang, Zhuoyang Chen, Yaliqin Yalikun, Lin He, Tiangang Liu, Gang Ma

**Affiliations:** 1 Bio-X Institutes, Key Laboratory for the Genetics of Developmental and Neuropsychiatric Disorders (Ministry of Education), Shanghai Jiao Tong University, No. 800 Dongchuan Rd., Shanghai, 200240 People’s Republic of China; 2 School of Life Sciences and Biotechnology, Shanghai Jiao Tong University, Shanghai, People’s Republic of China

**Keywords:** Taxol, Cyanobacteria, Cytochrome P450, Taxadiene-5α-ol, Transcriptomics

## Abstract

Taxol serves as an efficient natural anticancer agent with extensive applications in the treatment of diverse malignancies. Although advances in synthetic biology have enabled the de novo synthesis of taxol precursors in various microbial chassis, the total biosynthesis of taxol remains challengable owing to the restricted oxidation efficiency in heterotrophic microbes. Here, we engineered *Synechocystis* sp. PCC 6803 with modular metabolic pathways consisting of the methylerythritol phosphate pathway enzymes and taxol biosynthetic enzymes for production of taxadiene-5α-ol (T5α-ol), the key oxygenated intermediate of taxol. The best strain DIGT-P560 produced up to 17.43 mg/L of oxygenated taxanes and 4.32 mg/L of T5α-ol. Moreover, transcriptomic analysis of DIGT-P560 revealed that establishing a oxygenated taxane flux may enhance photosynthetic electron transfer efficiency and central metabolism in the engineered strain to ameliorate the metabolic disturbances triggered by the incorporation of exogenous genes. This is the first demonstration of photosynthetic production of taxadiene-5α-ol from CO_2_ in cyanobacteria, highlighting the broad prospects of engineered cyanobacteria as ﻿bio-solar cell factories for valuable terpenoids production and expanding the ideas for further rational engineering and optimization.

**Supplementary Information:**

The online version contains supplementary material available at 10.1186/s13068-024-02555-9.

## Introduction

Paclitaxel (Taxol) is a highly potent natural antineoplastic agent that exhibits remarkable efficacy against breast, ovarian, and pulmonary malignancies. It impedes the proliferation of neoplastic cells by arresting the cell cycle at the G2/M phase (the cell phase before mitosis) by stabilizing microtubule formation [1]. Presently, taxol production predominantly involves three methodologies: botanical extraction, chemical/semi-chemical synthesis, and biosynthesis. Taxol was initially derived from the bark of *Taxus brevifolia* [2]. Subsequently, researchers developed structural analogs with more extensive taxane frameworks from needles and employed chemically modified approaches for large-scale production [3]. Nonetheless, the current processes of plant extraction and semi-chemical synthesis depend heavily on natural resources and the cultivation industry. The utilization of ecologically sustainable and green biosynthetic strategies has the potential to comprehensively resolve the supply–demand incongruity of such bioactive agents. Furthermore, it has substantial strategic significance for research on natural-product production.

Numerous structural and regulatory genes involved in taxol biosynthesis have been identified [4]. The initial step in taxol biosynthesis is catalyzed by taxadiene synthase (TASY), which involves cyclization of the common diterpenoid precursor geranylgeranyl pyrophosphate (GGPP) to yield taxadiene and iso-taxadiene. Subsequently, the tricyclic structure of taxadiene undergoes oxidation and acylation at various sites through a series of cytochrome P450 oxidases (referred to hereafter as P450s) and acetyltransferases. Taxadiene-5α-ol (referred to hereafter as T5α-ol), the primary oxygenated taxane, is catalyzed by taxadiene-5α-hydroxylase (CYP725A4, referred to as T5αH hereafter), which introduces a hydroxyl group at the C5 position of taxadiene and transfers the double bond from C4(5) to C4(20) [5]. Meanwhile, a cytochrome P450 reductase (CPR) is required to ﻿donate two NADPH-derived electrons for the catalytic reaction [6].

With the rapid advancement of synthetic biology, metabolic engineering strategies, protein engineering, and multigroup techniques have found extensive applications in taxol biosynthesis research. To date, specific taxol precursors have been de novo synthesized in various microorganisms, including *Escherichia coli* [7–9] and *Sacharomyces cerevisiae* [10–12]. The highest reported yield of taxadiene in *E. coli* has reached to 1 g/L through a multivariate modular metabolic engineering strategy, although only 58 mg/L of T5α-ol was produced [7]. By balancing P450 module expression, engineering CPR interactions and modifying the N-terminus, Biggs et al. have increased the yield of total oxygenated taxanes to 570 mg/L in *E. coli* [8]. Another ambitious endeavor was made to introduce eight taxol biosynthetic genes simultaneously into *S. cerevisiae* for the biosynthesis of Baccatin III; however, only trace amounts of T5α-ol were detected [10]. Coupled with advanced biological process strategies, the engineered *S. cerevisiae* yields 34 mg/L of T5α-ol and 11 mg/L of taxadien-5α-yl-acetate [12], which represents the highest titers in yeast cell factories. However, it is still challenging to assemble the complex taxol biosynthesis pathway in *E. coli* because of the absence of internal membranes, which are essential for P450s integration [13, 14], and the inefficient regeneration of nicotinamide adenine dinucleotide phosphate (NADPH) might jeopardize the high activity of P450s in microbial chassis such as *E. coli* and yeast [14].

As prokaryotic and photoautotrophic microorganisms, cyanobacteria are considered as non-food-based feedstock resources that can convert solar energy and carbon dioxide for the synthesis of various carbon-based compounds [15]. Since cyanobacteria naturally synthesize numerous photosynthetic pigments such as carotenoids and chlorophylls [16], their native GGPP pools are supposed to be ample for stable supply of diterpene precusor, making them well suited for terpenoids production. On the other hand, P450s generally need to receive two electrons from NADPH or NADH to promote the catalytic reactions [14], the presence of photosynthetic electron transport chain in cyanobacteria ensures the regeneration of reducing power, thereby directly support the high activity of P450s. Most importantly, the successful integration of eukaryotic P450s into the photosynthetic thylakoid membranes has recasted cyanobacteria as promising hosts for P450s expression [17–19]. Given all that, cyanobacteria are competent candidates for the direct conversion of CO_2_ into valuable secondary metabolites such as terpenoids [20–22].

In this study, as a proof of concept, multiple endeavors were undertaken to achieve the efficient biosynthesis of various taxol precursors in the cyanobacterial model strain *Synechocystis* sp. PCC 6803, hereafter referred to as *Syn6803*. The taxadiene biosynthetic pathway was initially established in *Syn6803* by introducing heterologous GGPPS and TASY. By optimizing the upstream modules and increasing the biomass, the yield of taxadiene augmented approximately 11-fold. Subsequently, codon-optimized T5αH and its reductase partner CPR were integrated into the taxadiene-producing chassis with modular modifications implemented to enhance the efficiency of downstream oxygenated taxanes synthesis, including T5α-ol. Complemented by transcriptomic analysis, our aim was to elucidate the effects of introducing a heterologous taxol synthesis pathway on the intracellular energy and material metabolism of cyanobacterial chassis. Together, we sought to provide insights for establishing a more efficient photosynthetic platform for the direct conversion of CO_2_ into specialty compounds such as taxol and other terpenoids.

## Materials and methods

### Strain and cultivation conditions

All strains developed in this study were derived from *Synechocystis* sp. PCC 6803 wild type (hereafter described as WT). For flask shaking cultivation, the strains were grown at 30 °C in BG-11 medium [23] under continuous illumination of 25 µmol m^−2^ s^−1^, with shaking at 130 rpm for normal growth in a photoincubator shaker (ZQZY-AGS8, Zhichu, China). Genetically engineered strains were cultivated in BG11 medium containing the appropriate antibiotics (20 μg mL^−1^ spectinomycin, 20 μg mL^−1^ chloramphenicol and 50 μg mL^−1^ kanamycin). The initial inoculation concentration of the strains was OD_730_ = 0.2. Cell density was assessed by measuring the optiamal density at 730 nm using a Cary 60 UV spectrophotometer (Agilent, Germany). All strains were stored in BG-11 medium with 25% glycerol at − 80 °C.

For high density cultivation, the HDC 6.10B Starter Kit (CellDEG, Germany) was used to cultivate *Syn6803* cells according to the manufacturer’s instructions. The exact composition of the nutrient-enriched media, referred to as CD medium, is available in protocols.io (dx.doi.org/10.17504/protocols.io.2bxgapn) [24]. For taxanes production tests, HDC cultures were shaken at 200 rpm under continuous illumination of 100 µmol m^−2^ s^−1^ at 30 °C for the first two days. Afterwards, the light intensity was increased to 200 µmol m^−2^ s^−1^. Simultaneously, 1 mL overlay of dodecane (D221104, Sigma, USA) was added to 9 mL of cell cultures, and 1 mL of CD medium containing appropriate antibiotics was supplemented every 4 days to compensate the water evaporation.

### Plasmids construction

The host for plasmid construction was *E. coli* DH5α strain. All vectors, strains and primers used in this study are listed in Supplementary Tables S1 and S2. pBluescript II KS( +) purchased from Beijing qualityard biotechnology Co., Ltd. was used as a backbone. The target genes except for T5αH and CPR were amplified with standard PCR reactions using KOD-Plus-Neo high-fidelity DNA polymerase (KOD-401, Toyobo, Japan). The resulting fragments were assembled using Hieff Clone^™^ Plus Muiti One Step Cloning Kit (10912ES10, Yeasen, China) or via standard T4 DNA ligation reaction (15224025, Thermo Fisher Scientific, USA) according to the manufacturer’s instructions. All primers and genes were commercially synthesized by Suzhou Genewiz Inc. All plasmids constructed were verified by sanger sequencing (Genewiz Inc., China) and subsequently used for natural transformation.

### Chromosomal integration

For transformation of constructed vectors, 2 mL of fresh WT cells with an optical density (OD_730_) ranging from 0.4 to 0.6 were harvested by centrifugation at 2000*g* for 5 min and washed twice with ddH_2_O. The resulting cell pellet was resuspended in 400 μL fresh BG-11 medium. Subsequently, 5 μg plasmid was added to the cell mixture and incubated at 30 ℃ under constant light at an intensity of 25 µmol m^−2^ s^−1^ for 6–8 h. 200 μL of the cells was initially spread onto blank BG-11 agar plates for overnight incubation, then was transferred to the BG-11 agar plates supplemented with appropriate antibiotics (10 μg mL^−1^ spectinomycin, 10 μg·mL^−1^ chloramphenicol and 20 μg mL^−1^ kanamycin). After about 2 weeks, single clones appeared on the agars were screened by PCR verification to get positive clones. Several rounds of streaking were performed with increasing antibiotic concentrations to achieve complete segregation.

### Pigments extraction and measurement

After 72 h cultivation, 1 mL of cells from HDC cultivator were centrifuged (15,000×*g*, 7 min, 4 °C) and then resuspended in 1 mL of pre-cooled methanol. After shaking and mixing, the cell mixtures stand at 4 °C for 30 min, then were centrifuged (15,000*g*, 10 min, 4 °C), the organic phase containing chlorophyll a and phycocyanin was aliquoted to a 96-well plate. The OD value was detected at different wavelengths measured with Synergy2 Microplate Reader (BioTeK, USA), including 625 nm, 678 nm, 720 nm, and 750 nm. The following formulae were used for quantifications [25]:

For chlorophyll content: 14.94 (OD_678_–OD_750_)–0.616 (OD_720_–OD_750_).

For phycocyanin content: 0.138445 (OD_625_–OD_750_)–0.0354 (OD_678_–OD_750_).

All values were corrected using the optical density of the medium or methanol before calculation, and three replicates of the test were performed for each sample.

### Taxanes extraction and measurement

The productions of taxadiene and oxygenated taxanes were monitored by gas chromatography/mass spectrometry (GC/MS), using β-caryophyllene (75541, Sigma, USA) as an internal standard. The dodecane phase from different cell cultures was collected on the 10th day of cultivation. After centrifugation at 12,000 rpm at room temperature, the supernatant was diluted ten times in hexane containing 2.5 µg/mL β-Caryophyllene. Internal standards were prepared by diluting with hexane to concentrations between 0.625 and 50 μg/mL.

GC–MS analysis was conducted by using an Agilent Technologies 7890B GC System equipped with an Agilent 5977B mass-selective detector, a DB-5 ms capillary column (30 m × 250 μm × 0.25 μm, Agilent) was used for separation. Injections were made in pulsed splitless mode with an inlet temperature of 260 °C and pressure of 7.7758 psi. The GC method was following the method described by Biggs et al.[8]. The MS data were collected in the range of 45–550 *m*/*z* for each sample. Helium was used as the carrier gas at a flow rate of 1.0111 mL/min. The titer of taxanes were counted based on the peak area in comparison with the standard. GC-MS profiles depicting taxadiene and the four major oxygenated taxanes can be found in the Supplementary Figs. S1–S3.

### RNA-sequencing (RNA-Seq)

Two engineered *Syn6803* strains (DIGT, DIGT-P560) were grown up to the middle exponential phase and then collected by centrifugation. For each sample, three biological replicates were conducted to ensure reproducibility. Total RNA from 5 mL of HDC culture had been isolated using RNA isolater Total RNA Extraction Reagent (R401-01, Vazyme, China) following the modified method described by Schwarzkopf et al. [26]. Library construction and high-throughput sequencing were conducted by Shanghai Xu Ran Genomics Biotechnology Co. according to standard protocols. After sequencing, the raw data underwent initial processing, which involved the removal of adaptors, unknown bases, and low-quality reads. Subsequently, the filtered data were mapped to the *Synechocystis* reference genome in the NCBI Database (https://www.ncbi.nlm.nih.gov/datasets/genome/GCF_000009725.1/).

For gene expression analysis, raw sequence counts of known genes were quantified using the StringTie software. The expression levels of known genes were calculated using FPKM (Fragments Per Kilobase of transcript per million fragments mapped) metrics. Differential gene expression (DEG) analysis between different sample groups was performed using DESeq2 software, with the absolute value of log_2_FC ≥ 1 and *p* ≤ 0.05 to define DEGs. To gain full-scale insights into gene function, TopGO software was utilized for Gene Ontology functional analysis, which involved functional annotation and categorization of the entire gene set and the target gene set. In addition, pathway functional annotation and categorization of these genes were performed using KEGG functions.

## Results

### Introducing upstream pathway of taxol biosynthesis into *Synechocystis* chassis for taxadiene production

**Fig. 1:**
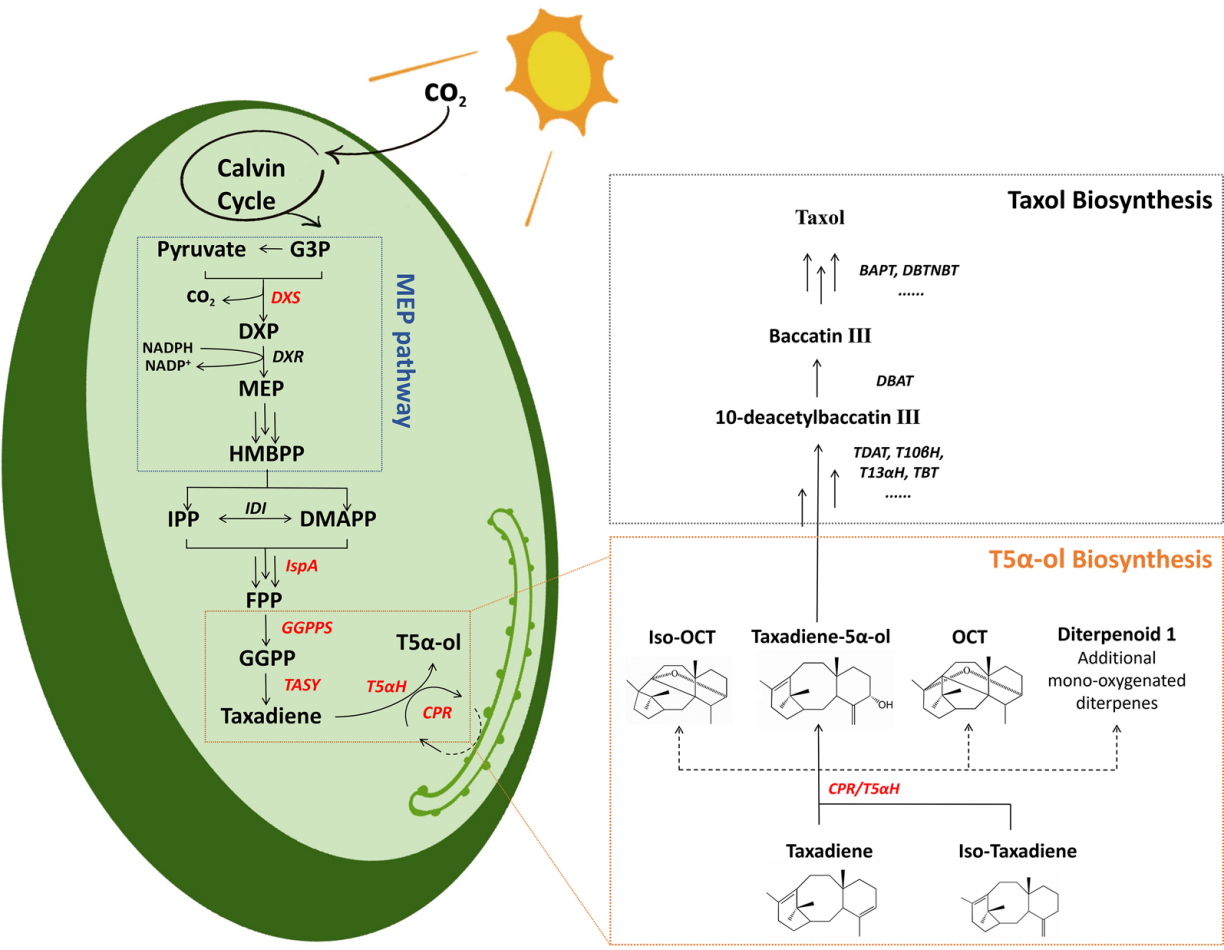
Engineered biosynthetic pathway in *Synechocystis* sp. PCC 6803. In our model cyanobacteria, the taxol heterologous synthesis pathway starts from the isoprenoid universal precursors IPP and DMAPP (provided by the native MEP pathway) to generate GGPP, then taxadiene and small amounts of its isomer iso-taxadiene. Under the catalysis of P450 oxidase (T5αH) and its cognate reductase (CPR), taxadiene is oxidized into a series of oxygenated taxanes (as shown in orange square), of which the specific product T5α-ol will undergo multiple rounds of stereospecific oxidations, acylations, and benzoylation to form Taxol (as shown in black dashed square). Genes highlighted in red represent heterologous genes that have been overexpressed through genome integration. G3P: glyceraldehyde-3 phosphate, DXP: deoxyxylulose 5-phosphate, MEP: methylerythritol 4-phosphate, HMBPP: (E)-4-Hydroxy-3-methyl-but-2-enyl pyrophosphate, IPP: isopentenyl diphosphate, DMAPP: dimethylallyl diphosphate, DXS: 1-deoxyxylulose-5-phosphate synthase, IspA: farnesyl phosphate synthase, GGPPS: geranylgeranyl diphosphate synthase, TASY: taxadiene synthase, T5αH: taxadiene-5α-hydroxylase, CPR: cytochrome P450 reductase

**Fig. 2:**
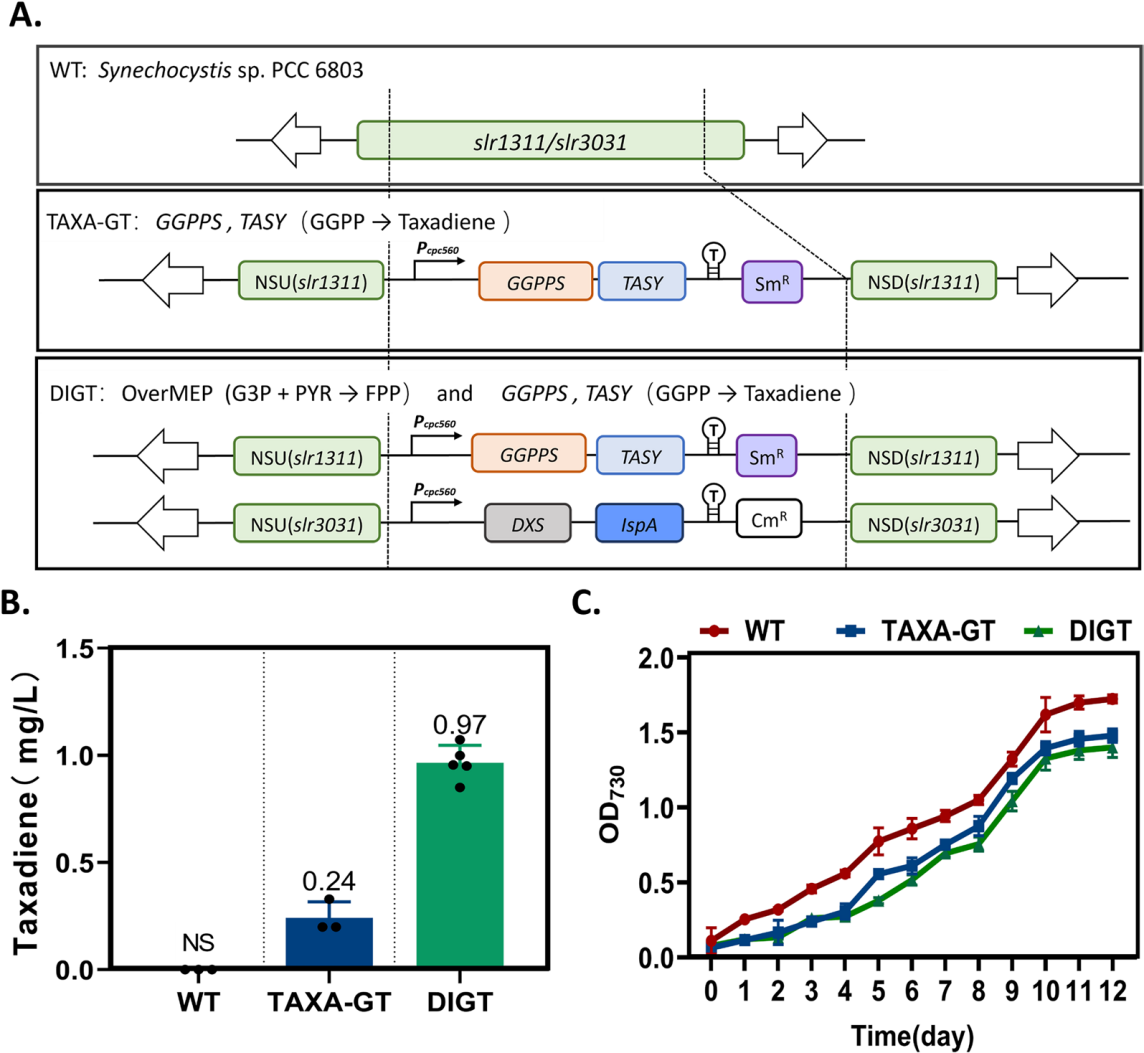
Metabolic engineering of* Synechocystis* sp. PCC 6803 for photosynthetic taxadiene production from CO_2_. **A** Schematic diagrams of the construction of *Syn6803* strains that produce taxadiene. The heterologous genes *dxs* and *ispA* derived from *E. coli* were introduced to a neutral site *slr3031*, while *ggpps* and *tasy* with truncation of plastid transport peptides were introduced to another site *slr1311*. **B** Taxadiene titers of Strains TAXA-GT and DIGT in shake flasks. **C** Biomass measured as absorbance at 730 nm of WT and engineered strains. All data are the mean ± standard deviation (SD) from triplicated shake flasks

In cyanobacteria, geranylgeranyl diphosphate (GGPP), a diterpene backbone comprising 20 carbon atoms, is originating from the condensation of three isopentenyl pyrophosphate (IPP) molecules and one dimethylallyl pyrophosphate (DMAPP) molecule via the methyl-D-erythritol 4-phosphate (MEP) pathway (Fig. 1). This pathway is prevalent in bacteria, cyanobacteria, green microalgae, and plant plastids [27]. As GGPP synthase (GGPPS) is recognized as the rate-limiting enzyme for GGPP production [28, 29], our initial step involved co-expression of heterologous GGPPS and TASY in the *Syn6803* wild-type strain to divert the metabolic flux from the MEP pathway toward taxol synthesis. The *ggpps* and *tasy* genes were cloned from plasmid pGB259 (Supplementary Table S1) and co-overexpressed as an operon under the strong promoter *P*cpc560 [30] and integrated into the *psbA2* locus (*slr1311*) of *Syn6803* genome [31, 32] through homologous recombination, creating the foundational engineered Strain TAXA-GT (Fig. 2A). To circumvent the solubility challenge of diterpenes in microbial cell cultures, engineered *Syn6803* cells were cultivated in liquid medium overlaid with dodecane, a biocompatible organic solvent that mitigates cellular toxicity [33]. This two-phase culture system is commonly employed to recover secreted hydrophobic products from cells [34, 35]. According to the taxadiene profiling results, Strain TAXA-GT produced approximately 0.24 mg/L of taxadiene after 7-day cultivation in shake flasks, while no trace of taxadiene was detected in the WT strain (Fig. 2B, Supplementary Fig. S1).

While the introduction of the mevalonate (MVA) pathway is often preferred over engineering the native MEP pathway in *E. coli* to enhance terpenoid production [34], the latter is more suitable for manipulation to benifit terpenoid biosynthesis in cyanobacteria. This is due to the relatively larger pools of glyceraldehyde-3-phosphate (GAP) and pyruvate compared to acetyl-CoA in cyanobacteria [36]. 1-deoxyxylulose-5-phosphate synthase (DXS) is generally considered as the key rate-limiting enzyme of the MEP pathway in various hosts [37, 38], while farnesyl phosphate synthase (IspA) is responsible for catalyzing the condensation of IPP and DMAPP to generate the terpenoid backbone molecule farnesyl diphosphate (FPP), plays a pivotal role in directing precursors toward the terpenoid synthesis pathway [21, 39] (Fig. 1). Therefore, the *dxs* and *ispA* genes cloned from *E. coli* were inserted between *slr2030* and *slr2031* (refered to as *slr3031*) [40] of Strain TAXA-GT to generate Strain DIGT (Fig. 2A). After 7-day cultivation, Strain DIGT accumulated up to 0.96 mg/L of taxadiene (Fig. 2B), which was four times the yield of Strain TAXA-GT. This result demonstrated that co-overexpression of DXS and IspA was beneficial for taxadiene biosynthesis by increasing the metabolic flux through the MEP pathway.

To assess whether the introduction of heterologous genes hindered cell growth, the cell densities of the WT, TAXA-GT, and DIGT strains were monitored for 12 d under normal conditions. The growth pattern of engineered strains exhibited a slghtly sluggish phenotype compared with WT (Fig. 2C). Given that alleviation of cytotoxicity caused by engineering the MEP pathway by overexpression of terpene synthase has been observed in both *E. coli* [34] and cyanobacteria [21], the growth defect of the taxadiene-producing strains suggests that maintenance of the metabolic flux balance between the MEP and terpenoid pathway is critical to preserve the robustness of the microbial host.

### Promoting the titer of taxadiene by high density cultivation

High-density cultivation (HDC) represents an efficient cultivation system designed specifically for cyanobacteria [24, 41]. By leveraging membrane-mediated CO_2_ supply technology, optimized nutrient-enriched CD medium, and intense illumination, HDC enables rapid and sustainable biomass accumulation. We harnessed this system to enhance biomass production and unlock its potential for generating ample taxol precursors in our engineered *Syn6803* strains.

**Fig. 3:**
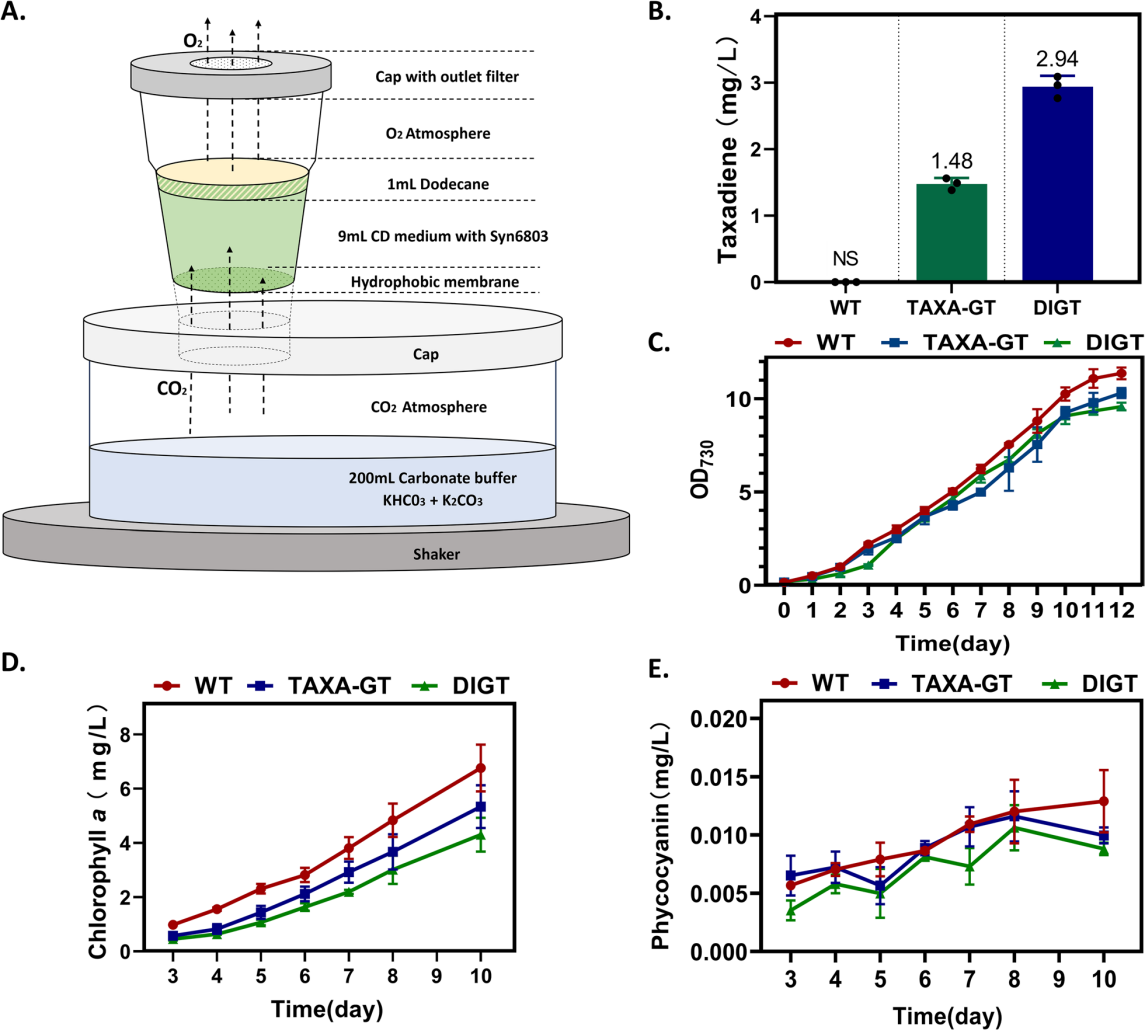
Growth characteristics and taxadiene production in High Density Cultivation (HDC) cultures. **A** Schematic representation of the HDC 6.10B system (CellDEG). **B** Taxadiene titers of Strains TAXA-GT and DIGT in HDC cultures. **C** Biomass measured as absorbance at 730 nm of WT and engineered strains. **D** and **E** Levels of chlorophyll *a* and phycocyanin contents from engineered cyanobacteria. All data are the mean ± standard deviation (SD) from triplicated HDC vessels

Strains WT, TAXA-GT, and DIGT were cultured in nutrient-rich CD medium within the HDC 6.10B device (CellDEG, Fig. 3A). In this setup, a concentrated KHCO_3_/K₂CO₃ buffer (the molar ratio of KHCO_3_ and K₂CO₃ was 9:1) in the container base continuously delivered 90 mbar of CO_2_-partial pressure, where the gas diffused through the hydrophobic membranes into turbulent cell suspensions. To maintain a homogenous culture, 9 mL of each culture was supplemented with 10% [*v*/*v*] dodecane overlay and agitated at 200 rpm under constant illumination with increasing light intensities.

By in situ extration and GC–MS analysis, taxadiene titers of 1.48 mg/L for the TAXA-GT strain and 2.94 mg/L for the DIGT strain were discovered in HDC media (Fig. 3B), which were increased by 5 times and two times, respectively, compared with shake flask culture. Cell density (OD_730_) and photosynthetic pigments content were recorded daily over a 10-day period ﻿in three independent cultivation runs. As Fig. 3C shown, the growth curve exhibited a similar trend to that of shake flask culture, with growth gradually slowing down after 10-day cultivation. The difference was that the growth rates of the engineered strains were close to that of WT before the sixth day, but then gradually lagged behind. Normally, the photosynthetic activity of cyanobacteria can be measured sideways by the content of photosynthetic pigment. There was a distinct difference in the chlorophyll *a* content between the WT and the engineered strain, especially for Strain DIGT which was almost half that of the WT (Fig. 3D). Phycocyanin is an important photosynthetic accessory pigment that transfers absorbed light energy from phycobilisomes to the chlorophyll-based photosynthetic apparatus [42]. The phycocyanin content in WT strain showed a steady upward trend with culture time, while that of the engineered strains fluctuated greatly and suddenly dropped after the 8th day (Fig. 3E), indicating that the photosynthetic efficiency of taxadiene producing strains may be disrupted in the later phase. Despite this, compared to shake flask, the growth rate of the engineered strains under HDC condition was not significantly different from that of the WT, suggesting that high light may be able to compensate to a certain extent for the adverse effects of insufficient photosynthetic pigments on photosynthesis. Given that the elevated biomass concentration will improve the efficiency of in situ extraction of terpenes, the HDC strategy was applied in subsequent experiments to introduce the downstream oxidation pathway of taxadiene.

### Combinatorial optimization of downstream pathway to produce oxygenated taxanes in *Synechocysis*

**Fig. 4:**
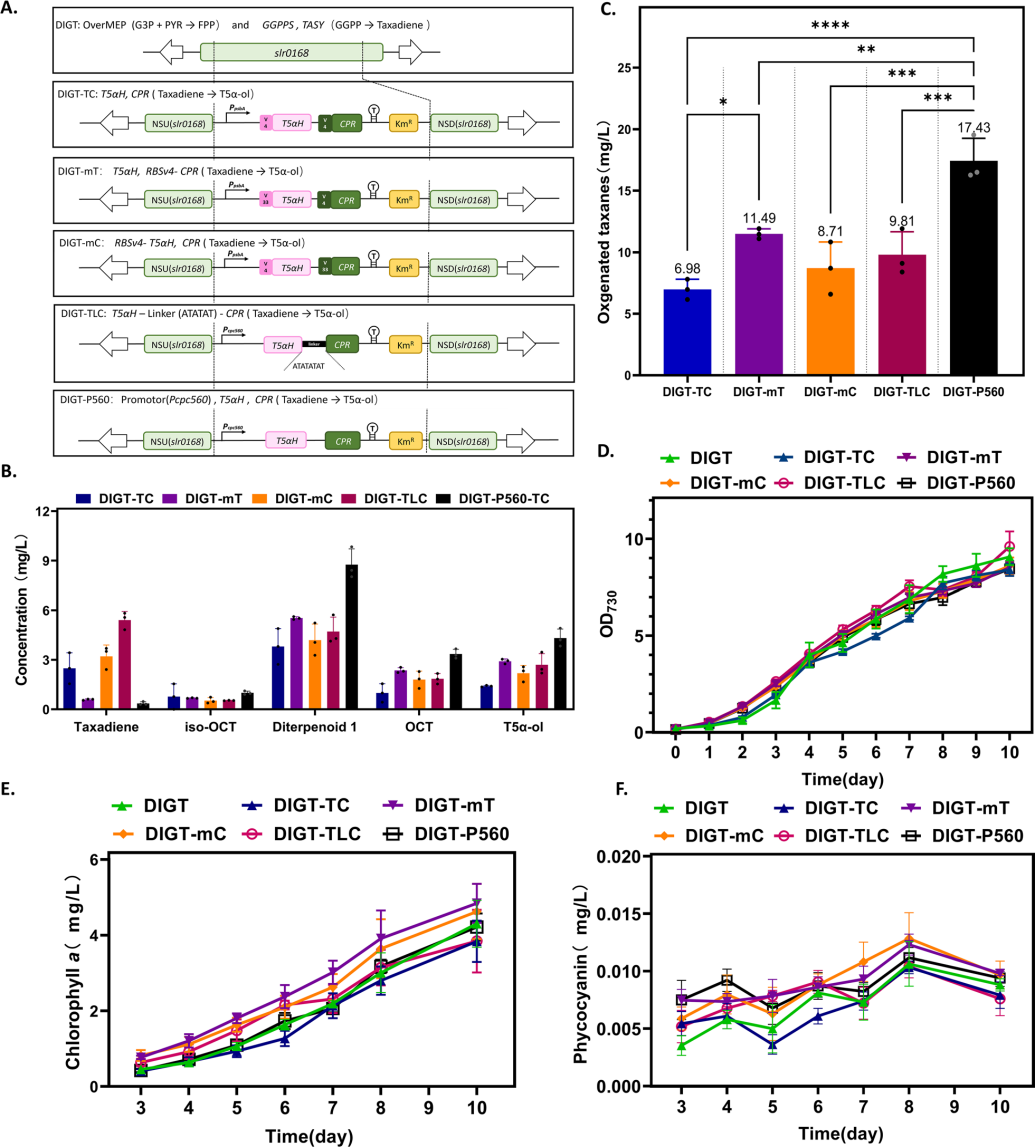
Combinatorial optimization of T5αH and CPR expression to produce oxygenated taxanes in *Syn6803*. **A** Schematic diagrams of the construction of *Syn6803* strains that produce oxygenated taxanes. The heterologous genes *t5αh* and *cpr* were codon-optimized and introduced together to the neutral site *slr0168* of taxadiene-producing strain DIGT. Five different plasmids were constructed with different combination of promoters, RBSs and linker to regulate the expression levels between *t5αh* and *cpr*. **B** The distribution of oxygenated taxanes produced in downstream strains. T5α-ol: Taxadiene-5α-ol. **C** Total oxygenated taxanes production in downstream strains. **D** Growth rate of DIGT and all downstream strains under high density cultivation, OD_730_ values were measured daily. **E** and **F** Levels of chlorophyll *a* and phycocyanin contents from engineered cyanobacteria. All data are the mean ± standard deviation (SD) from the triplicated culture, and ∗ in **C** represents statistical significance as indicated by Student’s *t*-test. ^∗^*p-*value of < 0.05; ^∗∗ ^*p-*value of < 0.01; ^∗∗∗^*p-*value of < 0.001; ^∗∗∗∗ ^*p-*value of < 0.0001

A pivotal step in engineering microbes for taxol production is the establishment of cytochrome P450s-based oxidation within the cellular environment. In previous studies conducted using *E. coli* chassis [7, 8], considering the absence of compartmentalization structures such as the endoplasmic reticulum in prokaryotes, the N-terminal domains of T5αH and CPR were engineered to ensure their proper function in cytoplasm. In this context, we introduced the intact coding sequences of T5αH and CPR (Supplementary Table S3) directly into the taxadiene-producing strain DIGT by employing codon optimization to enhance the expression efficiency of these heterologous proteins (Fig. 4A). The chromosomal loci *slr0168* [43] was used as the neutral site for expressing the target genes. It is well known that gene expression can be fine-tuned at the transcriptional level through promoter optimization or at the translational level by modifying ribosome-binding sites (RBSs). To facilitate genetic manipulation of *Syn6803*, a previous study established an RBS library consisting of 12 modified RBSs [44]. Notably, RBSv33 with a disrupted AT-rich region exhibited the highest expression level of the reporter gene, reaching 254% that of RBSv4, a relative strong RBS that was used to regulate the expression of ethylene forming enzyme in *Syn6803* [45]. Accordingly, we recruited RBSv4 and RBSv33 into our P450 expression module to investigate whether T5αH or CPR plays a more critical role in taxane oxygenation under the genetic background of *Syn6803*. Strains DIGT-TC, DIGT-mT and DIGT-mC harbored different combinations of RBSs under the regulation of a chimeric promoter *P*psbA* [44], while the expression of P450 module in Strain DIGT-P560 was under the control of *P*cpc560 (Fig. 4A). In addition, in the purpose of steering the metabolic flux toward taxane downstream pathway, T5αH was physically linked to its native CPR via a short flexible linker to create a chimera, resulting in Strain DIGT-TLC (Fig. 4A).

The product distributions of all downstream strains were determined by GC–MS analysis of the dodecane extracts. As shown in Supplementary Figs. S1 and S2, multiple taxane products were identified, including taxadiene and four major monohydroxylated compounds (parent ion at 288 m/z). Through comparison with gas chromatographs and mass spectra found in the literature [11], the peaks at 21.095, 21.378, 21.805, and 22.082 min were identified as iso-OCT, Diterpenoid 1, OCT, and T5α-ol, respectively (Supplementary Fig. S3). OCT and iso-OCT are considered as the major products in most T5αH-expressing hosts, such as *E. coli*, *S. cerevisiae*, and *Yarrowia lipolytica* [9, 46]*.* However, Diterpenoid 1, an isomer of T5α-ol, was emerging as the predominant oxygenated product in all downstream *Syn6803* strains (Fig. 4B), which was consistent with a recent work conducted in *S. cerevisiae* [11]. This result highlighted the significant influence of host organisms and cultivation conditions on the selectivity of T5αH. Simultaneously, T5α-ol was the second most abundant compound in all downstream strains, accounting for approximately 20–27% of the total products (Supplementary Table S4).

Considering the overall productivity of oxygenated taxanes, Strain DIGT-P560, featuring the “super strong” *P*cpc560 promoter, produced the highest titer of total oxygenated taxanes as 17.43 mg/L (Fig. 4C). Conversely, Strain DIGT-TC harboring the regulatory module of relatively low strength exhibited the lowest yield of 6.98 mg/L. It is noteworthy that a substantial amount of taxadiene was left over in the organic extracts of DIGT-TC, DIGT-mC, and DIGT-TLC (Supplementary Figs. S1, S2), suggesting the inefficiency of P450-mediated oxygenation toward taxadiene in those strains. In addition, Strain DIGT-mT, which might possess relatively higher T5αH expression than CPR resulted from the regulation of RBSv33 on T5αH, had produced more oxygenated products than Strain DIGT-mC that exhibited higher CPR expression (11.49 mg/L vs 8.71 mg/L). Taken together, it is plausible that a higher T5αH expression level could have advantageous effect on taxadiene oxygenation in *Syn6803*.

Compared with the basal Strain DIGT, the growth rates of some downstream strains transformed with T5αH/CPR were slightly lower (Fig. 4D), indicating that the expression of heterologous P450 enzyme may increase the metabolic burden of the cells. Interestingly, in terms of the synthesis of photosynthetic pigments, most downstream strains performed better than the upstream strain DIGT (Fig. 4E, F). It seems that the introduction of P450 enzyme improved the photosynthetic efficiency of the engineered strains to a certain extent. In conclusion, by manipulating the taxadiene downstream pathway via T5αH/CPR modular modification, we successfully developed an optimally engineered strain, DIGT-P560, which produced 17.43 mg/L of oxygenated taxanes and 4.32 mg/L of T5α-ol. While the rate of biomass and taxane accumulation may not currently rival that of heterotrophic microorganisms, efficient expression of the heterologous P450 enzyme led to a relatively high proportion of T5α-ol in our recombinant strains. This characteristic underscores the advantages of cyanobacterial chassis for further in vivo modification of the paclitaxel biosynthetic pathway when compared to other microbial hosts.

### Transcriptomic analysis for exploring the comprehensive effects of heterologous P450 expression on a whole-genome dimension of *Synechocystis*

**Fig. 5:**
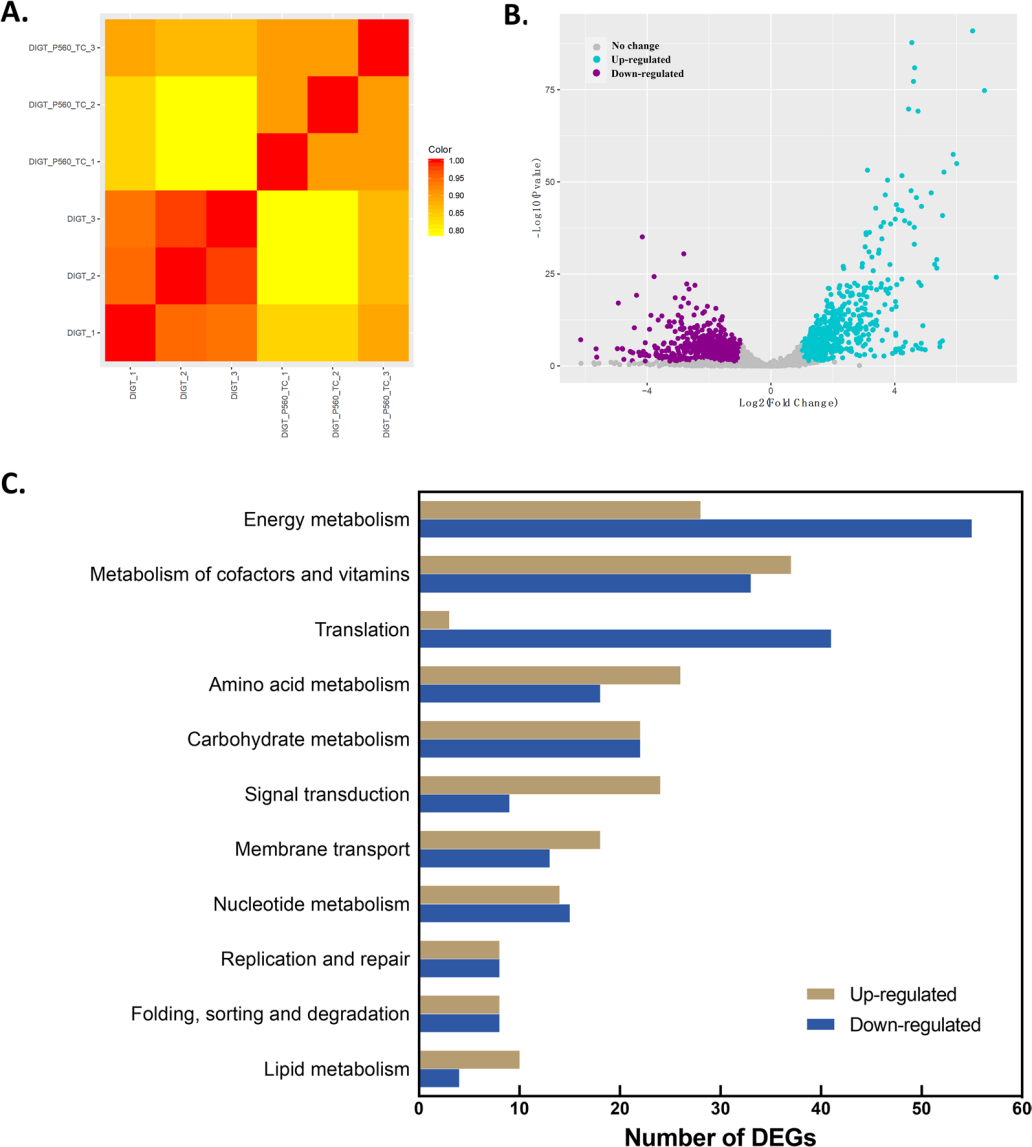
Differentially expressed genes (DEGs) analysis in downstream Strain DIGT-P560 relative to upstream Strain DIGT. **A** Pearson correlation heat map of all samples. **B** Volcano plots of differentially expressed genes in DIGT and DIGT-P560. Grey plot: Not DEGs; Blue plot: Up-regulated DEGs; Purple plot: Down-regulated DEGs. **C** DEGs number of up-regulated and down-regulated genes in annotated KEGG metabolic pathways. Light brown column: Up-regulated DEGs; Navy blue column: Down-regulated DEGs

To elucidate the potential mechanisms of unnatural diterponoid biosynthesis in cyanobacteria, a comprehensive whole-genome transcriptomic analysis was conducted to assess gene expression changes in DIGT and DIGT-P560 strains. Strains for RNA-seq were collected during the exponential growth phase. The Pearson Correlation Coefficient heat map shows the differences between samples within each group, allowing us to visually evaluate the similarities between biological replicates in the same group, as well as the differences between samples in different groups. As shown in Fig. 5A, there were significant differences between samples from DIGT and DIGT-P560 groups. The samples within the DIGT group were highly similar, while there were certain differences between the three replicates within the DIGT-P560 group. In comparison to the DIGT group, 1367 differentially expressed genes (DEGs) with Log2 of fold change (FC) ≥ 1 or ≤ -1 and *p* ≤ 0.05 were found in DIGT-P560 (Fig. 5B). With assignment to KEGG orthology database, apart from hypothetical and other functional genes, the rest DEGs were mainly categorized into 11 enriched pathways, including energy metabolism, metabolism of cofactors and vitamins, translation, amino acid metabolism, et al. (Fig. 5C). As expression of the majority of genes related to translation was significantly downregulated (Supplementary Data Set S1), the decreased levels of ribosomal proteins could potentially lead to disruption of ribosome assembly, which in turn may further diminish the translation efficiency of mature mRNAs in DIGT-P560 strains. Therefore, there were more downregulated genes than upregulated genes in DIGT-P560 (732 vs 635).

**Fig. 6:**
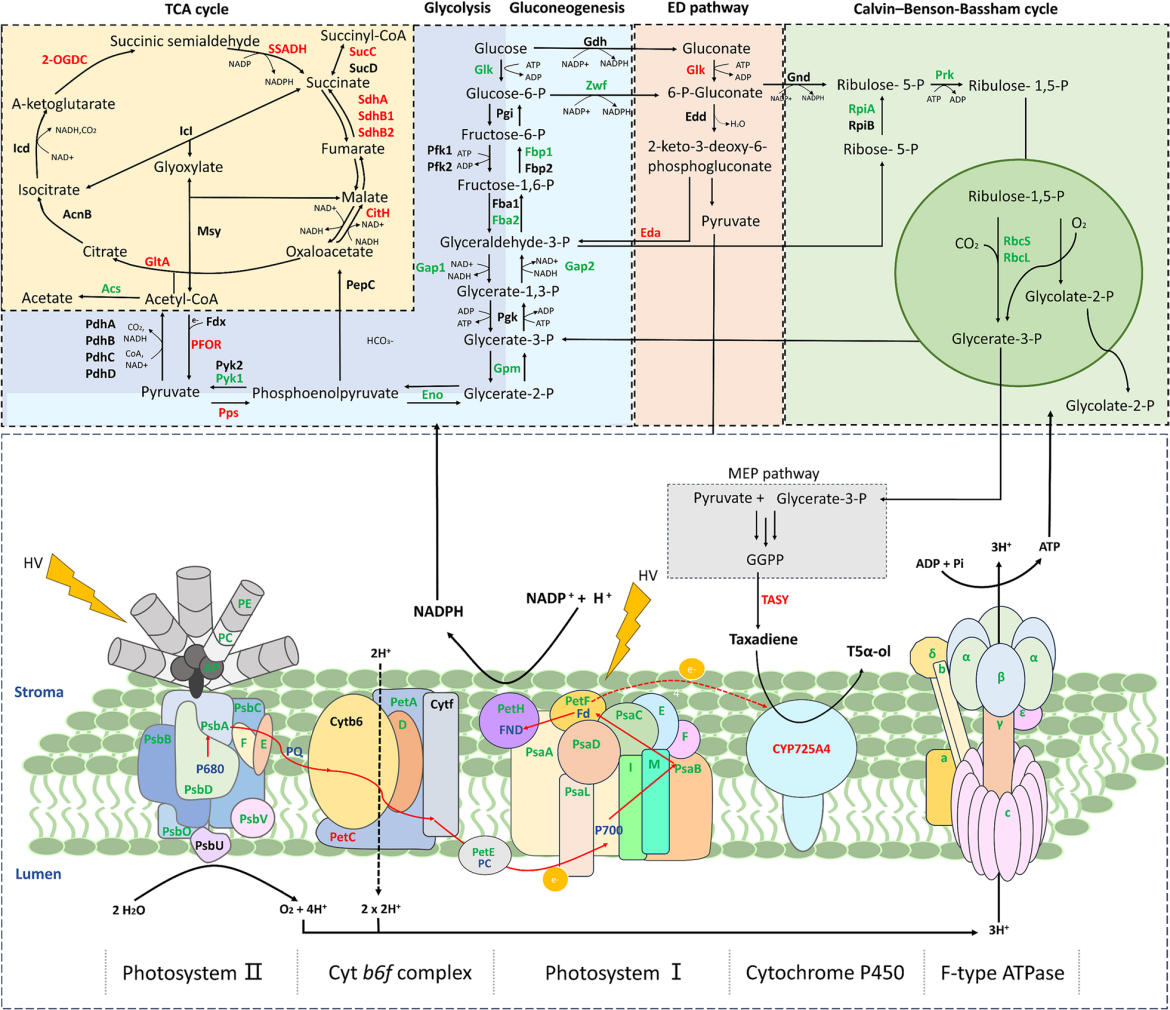
Comparative transcriptomic analysis of Strain DIGT-P560 and DIGT. Transcript abundance changes of the unigene involved in the TCA cycle (yellow panel), Glycolysis (navy blue panel), Gluconeogenesis (light blue panel), ED pathway (flesh panel), CBB cycle (green panel) and the photosynthetic chain (bottom panel). Up-regulated and down-regulated genes were labeled in red and green. TCA: tricarboxylic acid; ED: Entner-Doudoroff; CBB: Calvin–Benson–Bassham

For DIGT-P560, energy metabolism pathway had the largest number of DEGs, the expression of 80 related genes was significantly changed, of which nearly two-thirds were downregulated (Fig. 5C). The down-regulated genes were mainly concentrated in the photosynthesis, antenna proteins, and oxidative phosphorylation pathways (Fig. 6, Supplementary Data Set S1). This may be related to the fact that heterologous P450 enzymes were located on the thylakoid membrane, thereby affecting the expression of other photosystem proteins and F-type ATP synthase on the membrane. Meanwhile, the expression levels of genes related to biological processes such as nitrogen metabolism and sulfur metabolism showed an upward trend (Supplementary Data Set S1). It is noteworthy that the most significantly up-regulated genes in energy metabolism were *nifJ* (log_2_FC = 4.86) and *ppsA* (log_2_FC = 6.00), where *nifJ* is an oxidoreductase required for the transfer of electrons from pyruvate to flavodoxin, and *ppsA* is responsible for catalyzing the phosphorylation reaction of pyruvate [47]. The substantial up-regulation of these two genes suggested that the intracellular pyruvate content of DIGT-P560 could be higher than DIGT.

In cyanobacteria, carbohydrate metabolism typically includes central metabolic pathways, including glycolysis, the pentose phosphate pathway, the Calvin–Benson–Bassham (CBB) cycle, the Entner–Doudoroff (ED) pathway, and the tricarboxylic acid (TCA) cycle [47]. For Strain DIGT-P560, there were 44 DEGs belonging to carbohydrate metabolism, half of which were up-regulated (Fig. 5C) and mainly concentrated in the TCA cycle, pentose phosphate pathway and pyruvate metabolism (Supplementary Data Set S1). The TCA cycle, which is responsible for providing building blocks for growth, typically maintains a low flux rate under photoautotrophic conditions [48]. However, in DIGT-P560, five related genes were significantly upregulated, including *gltA*, *2-OGDC*, *SSADH*, *sdhA*, and *citH* (Fig. 6, Supplementary Data Set S1), suggesting the metabolic flux might be redirected to the TCA cycle to compensate the NADPH consumed by P450s, thereby maintaining the overall energy balance. In addition, the enhanced flux through the TCA cycle may lead to the increased synthesis of other essential compounds, such as amino acids and fatty acids, as evidenced by the higher number of upregulated genes related to amino acid and lipid metabolism in DIGT-P560 (Fig. 5C, Supplementary Data Set S1). Notably, lipids serve multiple crucial functions in cyanobacteria, including energy storage and structural support for cell membranes and thylakoid membranes [49]. In response to the challenges posed by the introduction of membrane-anchored proteins T5αH and CPR, DIGT-P560 may enhance its lipid synthesis capacity to maintain the normal function of thylakoids and provide a lipid-rich environment conducive to full activity of the P450 enzyme [6].

﻿When exploring the expression level of genes in signal transduction and membrane transport pathways, most genes were significantly upregulated in DIGT-P560 (Fig. 5C). Specifically, 22 out of 29 genes encoding two-component system proteins and 17 out of 26 genes encoding ATP-binding cassette (ABC) transporters showed upregulation (Supplementary Data Set S1). The two-component system is recognized for its tight regulation of ABC transporters, which play crucial roles in nutrient transport and cytoplasmic pH regulation [50, 51]. Both systems have the capacity to sense biotic and abiotic stresses as well as substrates such as peptides, amino acids, sugars, and antibiotics. Therefore, the activation of such genes in DIGT-P560 suggested that there were increased transmembrane material transport activities aimed at alleviating cellular stress in the taxane-producing strains. In addition, the expression levels of most genes related to DNA replication and repair, protein folding, sorting, and degradation were significantly higher in DIGT-P560 than in DIGT (Fig. 5C, Supplementary Data Set S1). This implied that cells under greater stress would have increased demands for restoration through processes such as DNA repair, homologous recombination, and RNA degradation.

## Discussion

As one of the most widely administered anticancer agents, the economical biosynthesis of taxol has remained a significant challenge for decades. While certain achievements have been made through heterologous expression of complex biosynthetic pathways in fast-growing microbial hosts, such as *E. coli*, *B. subtilis*, and *S. cerevisiae*, there has been no research on taxol biosynthesis by cyanobacteria despite their advantages of low culture cost, rapid growth, and ability to utilize CO_2_ as a carbon source. Therefore, we conducted a series of attempts in cyanobacteria model strain *Synechocystis* sp. PCC 6803 to explore a new platform for taxol biosynthesis.

The final conversion of taxadiene to taxol requires effective hydroxylation at eight specific sites: C1, C2, C4, C5, C7, C9, C10, and C13. The initial step involved introducing a hydroxyl group at the C5 position of taxadiene and relocating the double bond from C4(5) to C4(20) through the action of T5αH to yield T5α-ol [5]. This reaction is considered as the rate-limiting step in the entire oxygenation process. The native T5αH of *T. cuspidata* possesses a putative N-terminal sequence of 137 residues that is cleaved during maturation in plastids [52]. Truncating and modifying the N-terminal regions of T5αH have been found to promote the production of more active and soluble proteins in *E. coli*, addressing the challenges posed by the absence of internal membranes [7, 8]. In this study, the successful expression of intact T5αH and its reductase partner CPR clearly demonstrates the adaptability of the cyanobacterial chassis for the expression of N-terminally anchored cytochrome P450s, which will greatly facilitate further reconstruction of the complex taxol metabolic pathways in cyanobacterial cell factories.

In addition, it has been hypothesized that maintaining a balance between P450 and CPR is crucial for efficient catalysis of taxane substrates. Previous reports have shown that physically fusing P450/CPR does not provide advantages for protein expression or product synthesis [8], whereas the ratio of P450 to CPR in plant membranes is approximately 15:1 [53]. The oxygenated taxane profiles observed in our engineered strains were consistent with these findings. Strain DIGT-TLC exhibited the lowest oxygenated taxane productivity owing to the unsatisfied activity of the T5αH-CPR fusion protein. The concentration of total oxygenated taxanes, from highest to lowest, was observed in DIGT-P560, DIGT-mT, DIGT-mC, DIGT-TC, and DIGT-TLC, while the proportion of T5α-ol from highest to lowest was observed in DIGT-TLC, DIGT-mT, DIGT-mC, DIGT-P560, and DIGT-TC. These results indicate that manipulating the expression pattern of the T5αH/CPR combination at the translational or transcriptional level can significantly influence the productivity and distribution of downstream oxygenated products.

The activity of cytochrome P450s in the taxol pathway appears to be limited in prokaryotic hosts, as the production of taxadiene oxides significantly decreases when T5αH is introduced [7]. Although the delicate P450 module optimizations contribute to the effective enhancement of oxygenated taxanes in engineered *E. coli*, the titer of downstream products can not surpass their substrate, taxadiene [8]. Notably, in our study with *Syn6803*, even if the basal strain DIGT produced only 3 mg/L of taxadiene, the downstream strains transformed with the T5αH/CPR module produced up to 17 mg/L of oxygenated taxanes, significantly surpassing the substrate concentration. The transcriptomic analysis toward DIGT and DIGT-P560 indicated that, majority of downregulated DEGs were categorized into energy metabolism and translation, suggesting that the introduction of heterologous P450s could interfere with the photosystems and normal biological process of *Syn6803*. However, at the same time, certain genes were significantly upreglulated in DIGT-P560, particularly those related to the TCA cycle (Supplementary Data Set S1). Activation of such pathways may benefit the production of glyceraldehyde-3-P and pyruvate [47], providing more substrates for the MEP pathway to increase the GGPP pool for more efficient diterpene synthesis.

Although the photosystems of our engineered *Syn6803* strains were impaired owing to a significant reduction in the expression of related genes, the differences in growth rates between the wild-type and recombinant strains were relatively minor. How did these recombinant strains survive with their compromised photosystems? Phylloquinone is a prenylated naphthoquinone that plays a crucial role as an electron carrier in photosystem I and an electron acceptor for the formation of protein disulfide bonds [54]. Cyanobacterial strains lacking genes involved in phylloquinone biosynthesis, such as *menA*, *menB*, *menD*, and *menE*, have been shown to have slower photoautotrophic growth rates than the wild type [55, 56]. Plastoquinone (PQ), another important quinol, is located on the thylakoid membrane and forms a PQ pool with the reduced plastoquinone (PQH_2_) within the thylakoids. In cyanobacteria, PQ primarily mediates electron transfer between photosystem II and cytochrome b6f complex [57]. Our RNA-seq results revealed that genes related to phylloquinone (*menB*, *menC*, *menE*) and plastoquinone biosynthesis (*ubiC*) were significantly upregulated in DIGT-P560 (Supplementary Data Set S1). This discrepancy indicated a potential enhancement in the electron transport rate through the linear and cyclic photosynthetic electron transport chains. Meanwhile, several NAD biosynthetic genes were upregulated, such as *pntA/pntB* encoding pyridine nucleotide transhydrogenase (Supplementary Data Set S1), which catalyses electron transfer between NAD^+^ and NADP^+^ and regulates the NAD^+^/NADP^+^ ratio [58], thereby could provide sufficient reducing power for light-powered P450 activity. Intriguingly, previous studies have illustrated that P450s can serve as artificial electron sinks for excess electrons derived from photosynthesis and can increase the maximum rate of photosynthetic electron flow in cyanobacteria [13, 17, 59, 60]. It has been proved that cellular ATP and NADPH concentrations are directly proportional to P450 acitivity [13]. Our transcriptomic data may provide evidence of a potential mechanism for how the heterologous P450 electron sink influences photosynthetic electron transport and energy metabolism in cyanobacteria.

## Conclusion

In summary, our integrated approach led to the successful construction of a taxane-producing platform in photoautotrophic cyanobacteria. Given the flexibility of the metabolic network in this chassis, it is reasonable to expect that further improvements in the productivity of valuable oxygenated taxanes can be achieved through extensive research in the near future. For example, inducible promoters may be more suitable for heterologous cytochrome P450s enzymes to mitigate the metabolic burdens on the photosystems in cyanobacteria. Although the total biosynthesis of taxol in microbial cell factories remains a significant challenge, our work lays a solid foundation for the direct transformation of CO_2_ into taxol precursors in cyanobacteria in vivo. We hope that this will promote the widespread application of biosynthesis of various complex and valuable natural products in photosynthetic microorganisms.

### Supplementary Information

Supplemantary Material 1.

Supplementary Material 2.

## Data Availability

No data sets were generated or analysed during the current study.
