# Supplementary material for: Engineering cyanobacteria as a new platform for producing taxol precursors directly from carbon dioxide
Source: Biotechnol Biofuels Bioprod. 2024 Jul 16;17:99. doi: 10.1186/s13068-024-02555-9 (PMC11253407; doi:10.1186/s13068-024-02555-9)
Supplement: Supplementary file 2 — Supplementary Material 2. [file 13068_2024_2555_MOESM2_ESM.docx]

﻿ **Supplementary Information**

**Engineering cyanobacteria as a new platform for producing taxol precursors directly from carbon dioxide**

Jialing Zhong^1 #^, Yushu Wang^1 #^, Zhuoyang Chen^1^, Yaliqin Yalikun^1^, Lin He^1^, Tiangang Liu^2^, Gang Ma^1^

^1^Bio-X Institutes, Key Laboratory for the Genetics of Developmental and Neuropsychiatric Disorders (Ministry of Education), Shanghai Jiao Tong University, Shanghai, P.R. China

^2^School of Life Sciences and Biotechnology, Shanghai Jiao Tong University, Shanghai, P.R. China

To whom correspondence should be addressed:

Prof. Dr. Gang Ma

Key Laboratory for the Genetics of Developmental and Neuropsychiatric Disorders

Bio-X Institutes, Shanghai Jiao Tong University, Shanghai 200240, P.R. China

Tel : 0086-21-3420-7232

Email: [magang@sjtu.edu.cn](mailto:magang@sjtu.edu.cn)

**
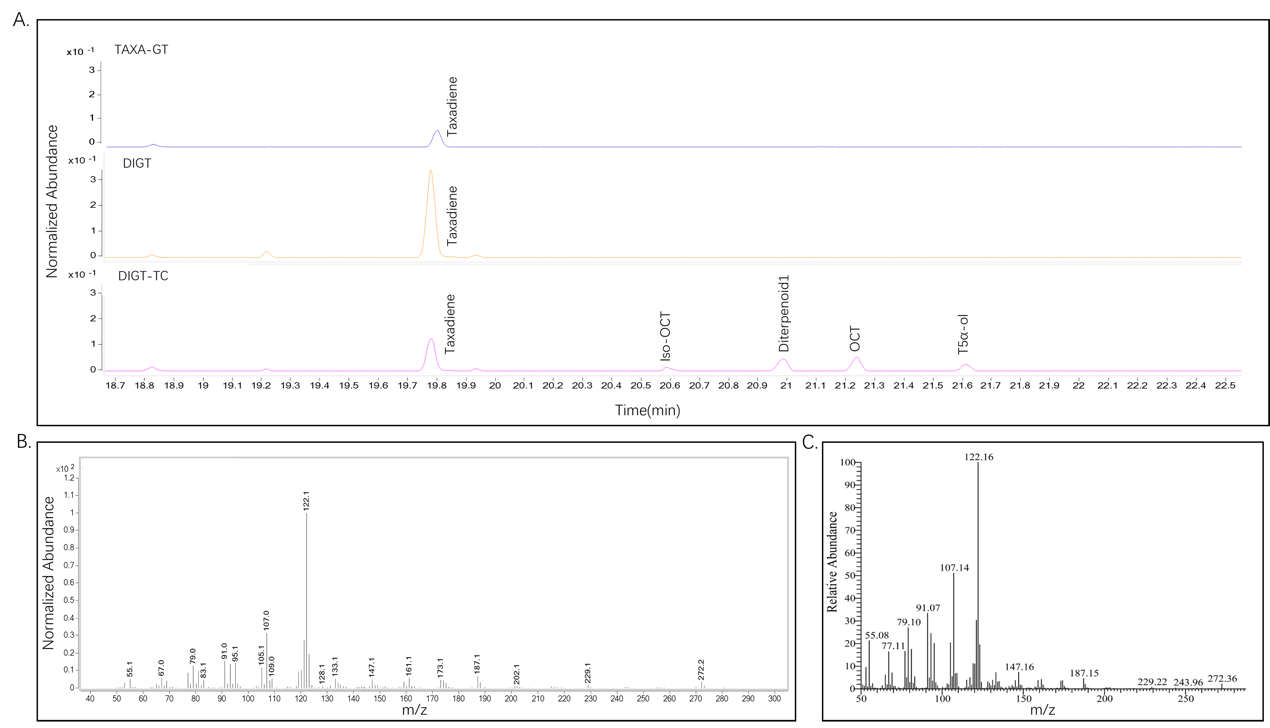
**

**Figure S1: ﻿Photosynthetic production of taxanes in Synechocystis sp. PCC6803.** In situ-extracted samples from dodecane overlay using engineered strains TAXA-GT, DIGT, and DIGT-TC were analyzed by GC–MS (A). Mass spectra of taxadiene from engineered strains (**B**) was comparable to the literature ^[1]^ (**C**).


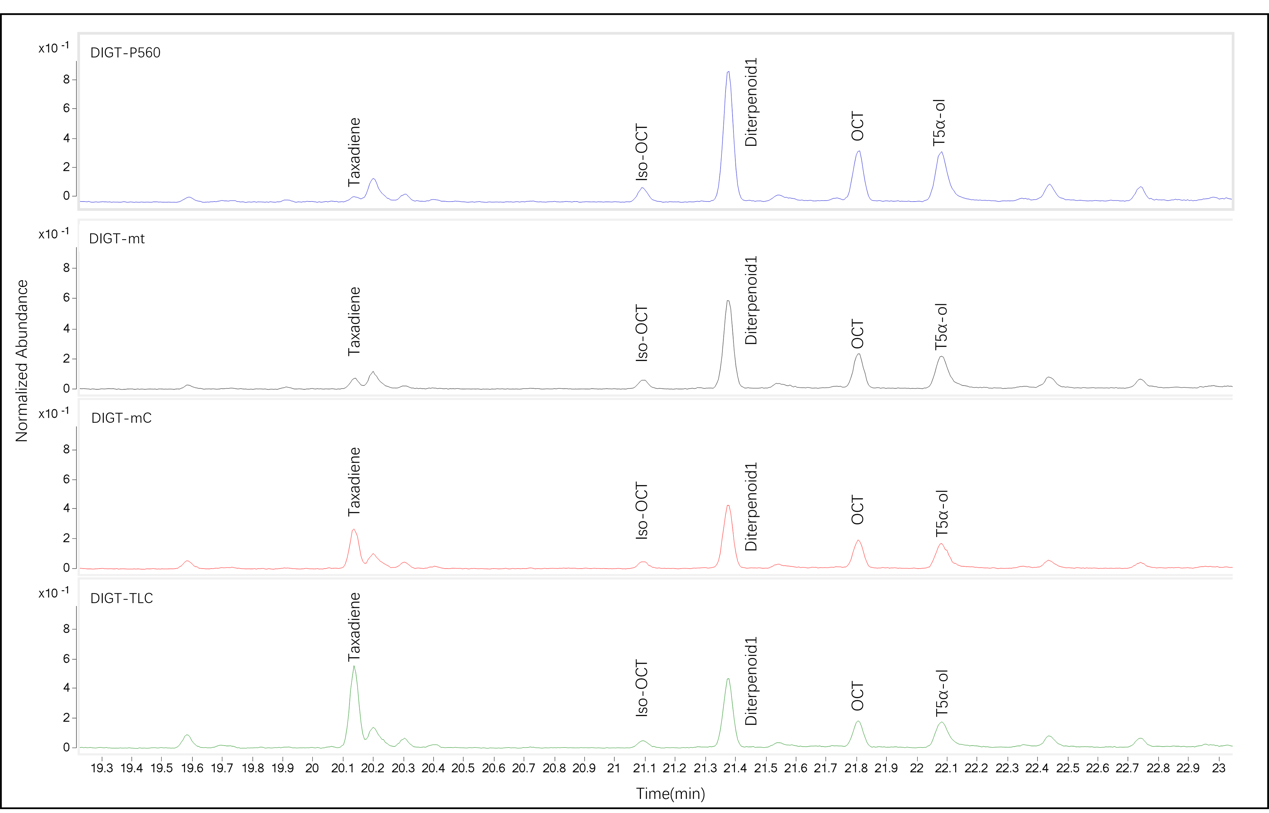


**Figure S2: Characteristic gas chromatograms with tentative identifications**. Chromatograms of different downstream strains showed the GC filtered for m/z 288 cultivated in CD medium using HDC 6.10B starter kit. ﻿The details of each peak and corresponding compounds can be found in Figure S3.


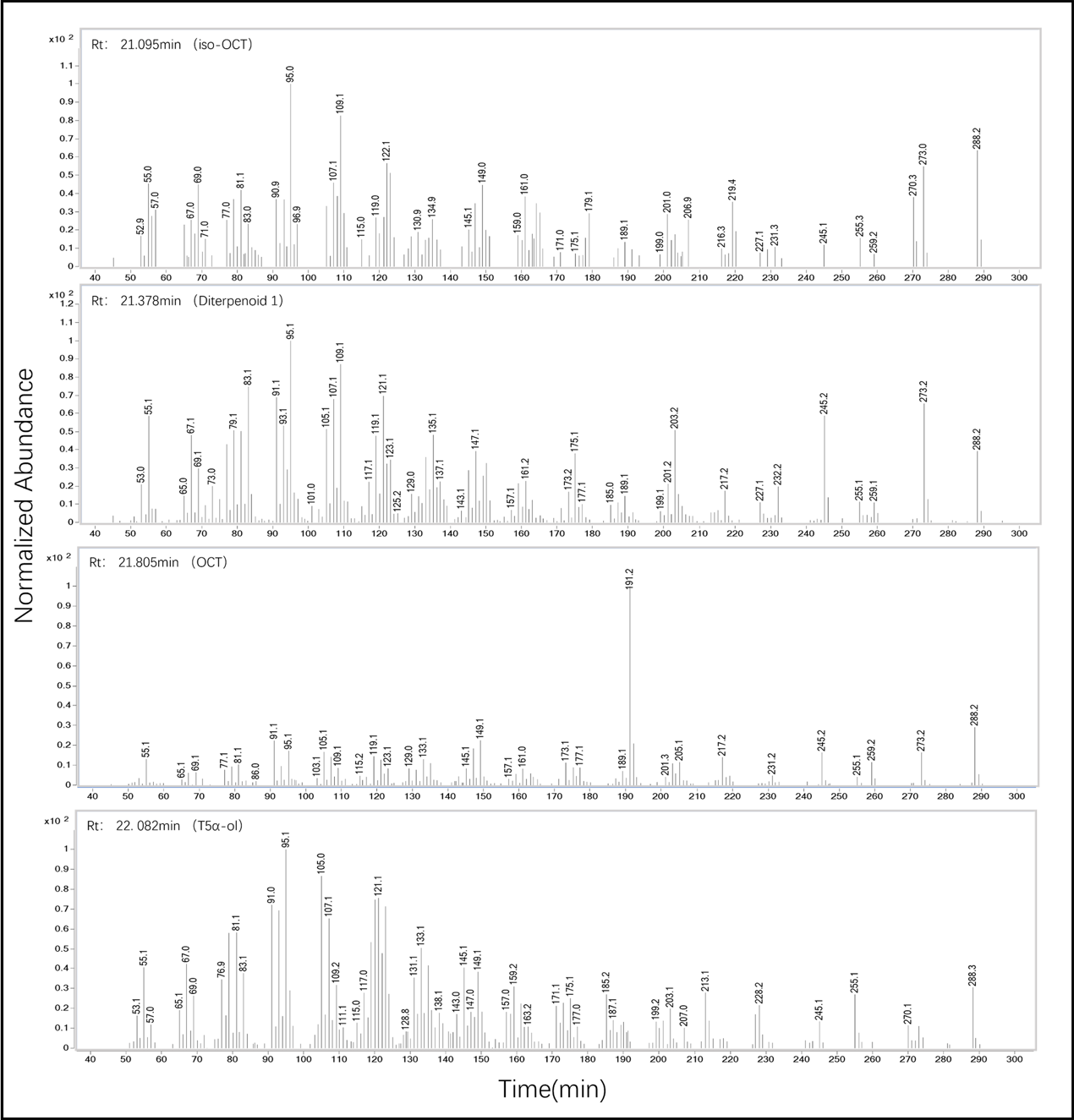


**Figure S3: Mass spectra for oxygenated taxanes produced by downstream strains.** All the products were identified through comparison to literature data [1]. ﻿GC-MS corresponds to iso-OCT for retention time 21.095 min. GC-MS corresponds to Diterpenoid 1 for retention time 21.378 min. GC-MS corresponds to OCT for retention time 21.805 min. GC-MS corresponds to ﻿taxadien-5α-ol for retention time 22.082 min.

**Table S1. Related vectors in this research**

| **Plasmid and strain** | **Characteristics** | **Reference** |
| --- | --- | --- |
| **Plasmids** |  |  |
| pGB259 | *P*T7: *tasy*, *ggpps*, *t5αh*, *cpr*, *Amp^R^*, pBR322 | This study |
| pCP3031-dxs-E | *P*cpc560: *dxs-E*, *Spe^R^*, *slr3031* targeting, pBR322 | ^[2]^ |
| pCP3031-ispA-E | *P*cpc560: *ispA-E*, *Spe^R^*, *slr3031* targeting, pBR322 | ^[2]^ |
| pBS1311 | Target gene overexpression, *Spe^R^*, *slr1311* targeting, pBR322 | This study |

| pBS0168 | Target gene overexpression, *Km^R^*, *slr0168* targeting, pBR322 | This study |
| --- | --- | --- |
| p1311-GGPPS-TASY | *P*cpc560: *ggpps*, *tasy*, *Spe^R^*, *slr1311* targeting, pBR322 | This study |
| p3031-dxs-ispA | *P*cpc560: *dxs-E*, *ispA-E*, *Cm^R^*, *slr3031* targeting, pBR322 | This study |
| p0168-T5αH-CPR | *P*psbA*: RBSv4-*t5αh*(co), RBSv4-*cpr*(co), *Km^R^*, *slr0168* targeting, pBR322 | This study |
| p0168-v4-T5αH-CPR | *P*psbA*: RBSv33-*t5αh*(co), RBSv4-*cpr*(co), *Km^R^*, *slr0168* targeting, pBR322 | This study |
| p0168-T5αH- v4-CPR | *P*psbA*: RBSv4-*t5αh*(co), RBSv33-*cpr*(co), *Km^R^*, *slr0168* targeting, pBR322 | This study |
| p0168-p560-T5αH- -CPR | *P*cpc560: *t5αh*(co), *cpr*(co), *Km^R^*, *slr0168* targeting, pBR322 | This study |
| p0168-p560-T5αH- Linker-CPR | *P*cpc560: *t5αh*(co)**cpr*(co), *Km^R^*, *slr0168* targeting, pBR322 | This study |
| **Strains** |  |  |
| WT | *Synechocystis* sp. PCC 6803 | ATCC |
| TAXA-GT | *P*cpc560: *ggpps-tasy* :: *Spe^R^* | This study |
| DIGT | TAXA-GT + *P*cpc560: *dxs-E-ispA-E* :: *Cm^R^* | This study |
| DIGT-TC | DIGT-TC + *P*psbA*: RBSv4-*t5αh*(co)-RBSv4-*cpr*(co) :: *Km^R^* | This study |
| DIGT-mT | DIGT-TC + *P*psbA*: RBSv33-*t5αh*(co)-RBSv4-*cpr*(co) :: *Km^R^* | This study |
| DIGT-mC | DIGT-TC + *P*psbA*: RBSv4-*t5αh*(co)-RBSv33-*cpr*(co) :: *Km^R^* | This study |
| DIGT-TLC | DIGT-TC + *P*cpc560: *t5αh*(co)**cpr*(co) :: *Km^R^* | This study |
| DIGT-P560 | DIGT-TC + *P*cpc560: *t5αh*(co)-*cpr*(co) :: *Km^R^* | This study |

**Table S2. Primers used in this research**

| **Name** | **Sequence (5’-3’)** |
| --- | --- |
| XhoI-1311_Pcpc_F | CCGCTCGAGACCTGTAGAGAAGAGTCCCTGA |
| 1311_Pcpc_R-HindIII | GGCAAGCTTTGAATTAATCTCCTACTTGACTTT |
| PstI-0168_Pcpc_F | AAAACTGCAGACCTGTAGAGAAGAGTCCCTGA |
| 0168_Pcpc_R-XbaI | CTAGTCTAGATGAATTAATCTCCTACTTGACTTT |
| HindIII-His-GGPPS_F | CCGAAGCTTATGCATCATCATCATCATCATTTTGATTTCAATGAATATATGAAAAGTAAGGC |
| GGPPS_R-EcoRI | CCCGAATTCTCAGTTCTGACGAAACGCA |
| EcoRI-RBS-HA-TASY_F | CCCGAATTCAGGAGGTAATATATGTACCCATACGATGTTCCAGATTACGCTTCTAGCTCTACGGGTACGTC |
| TASY_R-Term-SpeI | CTAGACTAGTAAAAAAAACCCCGCCCTGTCAGGGGCGGGGTTTTTTTTTTTAGACCTGGATTGGATCGATG |
| XbaI-Flag-P450_F | CTAGTCTAGAATGGATTACAAGGATGACGACGATAAGGCTCTGTTATTAGCAGTTTT |
| P450_R-HindIII | GGCAAGCTTTTACGGACGAGGGAACAG |
| HindIII-RBS-Myc-CPR_F | CCGAAGCTTAGGAGGTAATATATGGAGCAGAAACTCATCTCTGAAGAGGATCTGGCTCTGTTATTAGCAGT |
| CPR_R-Term-NotI | TAAGAATGCGGCCGCAAAAAAAACCCCGCCCTGTCAGGGGCGGGGTTTTTTTTTTTACCAAATATCCCGTAAGTAG |
| XbaI-NotI-0168_F | TGCTCTAGAGCGGCCGCGTTATAAAATAAACTTA |
| 0168-PstI_R | AAAAACTGCAGGTCCCCTTTGAGGATAATG |
| EcoRI-SpeI-1311_F | CCGGAATTCACTAGTTTTAAAGGAGGTTAACAAT |
| 1311-XhoI_R | GGCCTCGAGATTTTTTGGTCACATTGTC |
| V0168-F | GGCCGCGTTATAAAATAAACTTAACAAATCTATACCC |
| V0168-R | TGCAGGTCCCCTTTGAGGATAATGTCG |
| gb-T5aH-F | CGACATTATCCTCAAAGGGGACCTGCAAACACCACTGGGCCTACT |
| gb-CPR-R | GGGTATAGATTTGTTAAGTTTATTTTATAACGCGGCCCTACAGATCCTCTTCAGAGATG |
| mut-T5V33-F | CAAGATTTACCGTTCCCATAAAGAAGGAGAAACAGCATGGATGCTTTATACAAATCTACAGTTGC |
| mut-T5V33-R | GGGAACGGTAAATCTTGTTATCTTGTTATTCAACAGTATAACATGTCTTATACGCCCG |
| mut-CPRV33-F | CAAGATTTACCGTTCCCATAAAGAAGGAGAAACAGCATGCAAGCTAATTCTAATACAGTTGAGGG |
| mut-CPRV33-R | GGGAACGGTAAATCTTGTTATTTACTTATCGTCGTCATCCTTGTAATCAGG |
| P560-T5-F | CACTCGAGAAGATCTTGATATGGATGCTTTATACAAATCTACAGTTGC |
| T5-V0168-R | CCATATCAAGATCTTCTCGAGTG |
| T5-CPR-F | AGGAGGTAATATATGCAAGCTAATTCTAATACAGTTGAGGG |
| CPR-T5-R | GCTTGCATATATTACCTCCTTTACTTATCGTCGTCATCCTTGTAATC |
| CPR-V0168-F | GAAACTCATCTCTGAAGAGGATCTG |
| Ter-CPR-R | CAGATCCTCTTCAGAGATGAGTTTCTGCT |
| L-CPR-F | GACCTGGTTCAACAGGTTCACAAGCTAATTCTAATACAGTTGAGGG |
| L-T5-R | TGAACCTGTTGAACCAGGTCTAGGGAACAGCTTTATTGAG |
| TASY_test_F | CTGGAAGGGCTGGAAGAGGTTAT |
| TASY_test_R | TCAAAGGTCGCCGAGGAGAAGTA |
| GGPPS_test_F | AAAGTAAGGCTGTTGCGGTAGACG |
| GGPPS_test_R | TTGCCAAGCTCTTCGCTGGATTT |
| slr1311_ F | GAGCCGTTGCTGGTTCTT |
| slr1311_R | ACCGACCAAAGTAGCCGTG |
| slr0168_F | AAATGGTGGGACCCAACGG |
| slr0168_R | CTTCGGAGGGCAAAGCAAC |
| 3031_F | CTGTCTCCCGCCATTGCTA |
| 3031_R | ATGGCCCCCAACAGATCCT |
| CT-test-F | GTATGGCTGCTTGTTTGCGT |
| CT-test-R | AGCCATCGGAGGAAATGGTG |

**Table S3. The﻿ codon optimized sequences of T5αH and CPR**

| Protein | Nucleotide sequence |
| --- | --- |
| Taxadiene 5α-hydroxylase (T5αH) | ATGGATGCTTTATACAAATCTACAGTTGCTAAATTTAATGAGGTTACACAATTAGATTGCTCTACAGAGTCCTTCTCGATCGCGCTATCCGCCATAGCGGGAATCCTGCTCTTATTGCTATTGTTCCGATCTAAGCGACATTCTTCTTTAAAGCTGCCGCCTGGGAAATTAGGGATACCGTTTATTGGAGAATCCTTTATATTCTTACGTGCGCTACGATCAAACAGTCTCGAACAGTTCTTCGACGAACGGGTAAAGAAGTTCGGGCTAGTATTCAAGACGTCATTAATTGGGCACCCTACAGTTGTGCTTTGTGGGCCTGCTGGGAATAGATTAATCCTGTCCAACGAAGAGAAGTTGGTCCAAATGTCTTGGCCTGCTCAATTTATGAAATTAATGGGTGAGAATTCCGTTGCTACAAGAAGAGGAGAAGACCACATTGTTATGAGATCTGCTTTAGCTGGGTTCTTCGGACCCGGTGCGCTGCAATCTTACATTGGGAAGATGAACACAGAGATTCAATCTCACATTAATGAGAAATGGAAAGGGAAGGACGAAGTAAACGTACTCCCTCTTGTTCGCGAACTAGTGTTTAATATTTCTGCCATCCTGTTCTTCAACATCTACGATAAGCAGGAACAGGACCGTCTTCATAAGCTTCTTGAAACAATCTTGGTCGGGTCTTTCGCATTGCCTATTGATTTACCTGGGTTTGGGTTCCATCGGGCACTTCAGGGACGCGCCAAGCTAAACAAGATAATGCTATCACTGATCAAGAAACGCAAGGAGGATTTACAATCTGGGAGTGCCACCGCAACTCAGGACCTACTTTCCGTTCTACTAACCTTCAGAGACGACAAGGGCACTCCTTTAACGAACGACGAAATCCTCGATAACTTCTCTTCATTATTACACGCTAGTTATGACACGACGACCAGCCCGATGGCCCTTATCTTTAAACTGCTCTCTTCTAATCCTGAGTGCTACCAGAAGGTAGTCCAAGAGCAATTAGAGATTCTGAGTAACAAGGAAGAGGGTGAAGAAATTACATGGAAAGATTTAAAGGCCATGAAATACACATGGCAAGTTGCTCAAGAGACATTAAGAATGTTTCCTCCTGTGTTCGGTACTTTCCGTAAAGCCATCACAGATATTCAATACGATGGGTACACAATTCCTAAAGGGTGGAAATTATTATGGACAACATACTCTACACACCCTAAAGATTTATACTTTAATGAGCCTGAGAAATTTATGCCTTCTAGATTTGATCAAGAGGGTAAGCATGTTGCTCCTTACACATTCCTACCCTTCGGCGGCGGGCAACGGTCTTGCGTTGGGTGGGAGTTCTCCAAGATGGAGATCCTGTTATTCGTACACCACTTTGTTAAGACTTTCTCGTCGTACACACCTGTTGATCCTGATGAGAAGATCAGTGGCGACCCATTACCTCCTTTACCTTCTAAAGGGTTCTCAATAAAGCTGTTCCCTAGACCT |
| ﻿﻿Cytochrome P450 reductase (CPR) | CAAGCTAATTCTAATACAGTTGAGGGTGCCAGTCAAGGGAAGTCCCTACTGGACATATCTAGATTAGATCACATCTTCGCCTTGCTCCTAAATGGGAAAGGAGGAGACCTGGGCGCCATGACGGGCAGTGCACTAATTCTAACTGAGAATAGTCAGAACCTTATGATCCTTACCACGGCGCTTGCGGTATTAGTAGCTTGCGTCTTCTTCTTCGTCTGGAGAAGAGGAGGCTCAGACACACAGAAGCCCGCAGTTAGACCTACACCTTTAGTTAAAGAGGAGGATGAGGAGGAGGAGGATGATTCTGCTAAGAAGAAGGTGACGATATTCTTCGGAACTCAAACAGGGACAGCTGAGGGTTTCGCAAAGGCCCTTGCTGAGGAGGCTAAAGCTAGATACGAGAAAGCTGTATTCAAGGTGGTTGATTTAGATAATTACGCTGCTGATGATGAGCAATACGAGGAGAAATTAAAGAAGGAGAAACTTGCTTTCTTCATGCTCGCTACATACGGAGACGGCGAACCCACAGATAATGCTGCTAGATTCTATAAGTGGTTCCTGGAAGGTAAGGAAAGAGAGCCTTGGTTATCTGATTTAACATACGGTGTCTTCGGACTCGGGAATAGACAATACGAGCACTTTAATAAAGTTGCTAAAGCTGTTGATGAGGTACTGATCGAGCAAGGAGCGAAACGTCTCGTTCCTGTTGGGTTAGGCGACGACGATCAATGCATTGAGGATGATTTCACTGCATGGAGAGAGCAAGTTTGGCCTGAGTTAGATCAATTATTAAGAGATGAGGATGATGAGCCTACATCTGCTACACCTTACACAGCTGCTATTCCTGAGTACAGAGTTGAGATTTACGATTCTGTTGTTTCTGTTTACGAAGAAACTCATGCGCTGAAGCAGAACGGGCAAGCTGTTTACGATATTCACCACCCTTGCAGATCTAATGTTGCTGTTAGAAGAGAGTTACACACACCTTTATCTGATAGATCTTGCATTCACTTAGAGTTTGATATTTCTGATACAGGGTTAATTTACGAGACAGGTGACCATGTTGGTGTGCATACCGAGAATTCTATAGAAACAGTTGAGGAGGCTGCTAAATTATTAGGGTACCAATTAGATACAATCTTCAGTGTCCACGGAGACAAGGAGGATGGGACACCTTTAGGTGGCAGTTCCTTACCTCCTCCTTTCCCGGGTCCTTGCACATTAAGAACTGCACTTGCGCGTTATGCTGATTTATTAAATCCTCCTAGAAAGGCCGCATTCTTGGCGTTAGCTGCTCACGCTTCTGATCCTGCTGAGGCTGAGAGATTAAAGTTTCTGTCGTCTCCTGCTGGGAAAGATGAGTACTCTCAATGGGTTACAGCTTCTCAAAGATCCTTGCTCGAAATAATGGCTGAGTTTCCTTCTGCTAAACCTCCTTTAGGTGTCTTCTTTGCGGCAATTGCTCCTAGATTACAACCTAGATACTACTCTATTTCTTCTTCTCCTAGATTTGCTCCTTCTAGAATTCACGTTACATGCGCTTTAGTTTACGGGCCTTCTCCTACAGGGAGAATTCACAAAGGCGTATGTTCTAATTGGATGAAGAACTCCTTACCTTCTGAAGAAACCCATGACTGTTCTTGGGCTCCTGTCTTCGTGCGCCAATCTAATTTCAAGCTACCTGCTGATTCTACAACACCTATTGTTATGGTTGGGCCTGGGACAGGGTTTGCTCCTTTCCGCGGGTTTCTCCAGGAGAGAGCTAAATTACAAGAGGCTGGAGAGAAACTGGGTCCCGCGGTATTGTTCTTTGGCTGTAGAAATAGACAAATGGATTACATTTACGAGGATGAGTTAAAGGGTTATGTTGAGAAAGGGATCCTTACGAATTTAATTGTTGCTTTCAGCCGTGAGGGTGCAACTAAGGAATATGTTCAACACAAGATGCTAGAGAAAGCTTCTGATACATGGTCTTTAATTGCTCAAGGAGGTTATCTTTACGTTTGCGGCGACGCCAAAGGGATGGCTAGAGATGTTCACAGAACATTACACACAATTGTTCAAGAGCAGGAATCGGTCGACAGCTCCAAGGCCGAATTCTTAGTCAAGAAGCTACAAATGGATGGGAGATACTTAAGAGATATTTGG |

**Table S4. The distribution of different oxygenated taxanes in engineered strains**

| Strain | Percentage (%) | | | |
| --- | --- | --- | --- | --- |
|  | Iso-OCT | Diterpenoid 1 | OCT | Taxadiene-5α-ol |
| DIGT-TC | 11.06 | 54.58 | 14.22 | 20.14 |
| DIGT-mT | 5.94 | 48.13 | 20.56 | 25.37 |
| DIGT-mC | 6.09 | 48.1 | 20.67 | 25.14 |
| DIGT-TLC | 5.47 | 48.15 | 18.85 | 27.53 |
| DIGT-P560 | 5.71 | 50.25 | 19.25 | 24.79 |

**Dataset S1**: Categorized DEGs in Strain DIGT-P560.

**References**

1. Walls LE, Malci K, Nowrouzi B, Li RA, d'Espaux L, Wong J, Dennis JA, Semiao AJC, Wallace S, Martinez JL *et al*: **Optimizing the biosynthesis of oxygenated and acetylated Taxol precursors in Saccharomyces cerevisiae using advanced bioprocessing strategies**. *Biotechnol Bioeng* 2021, **118**(1):279-293.

2. Diao J, Song X, Zhang L, Cui J, Chen L, Zhang W: **Tailoring cyanobacteria as a new platform for highly efficient synthesis of astaxanthin**. *Metab Eng* 2020, **61**:275-287.
